# Orbital Swelling and Ptosis as an Initial Presentation of Childhood Acute Lymphoblastic Leukaemia: A Case Report and Review of the Literature

**DOI:** 10.1155/2021/5587767

**Published:** 2021-06-01

**Authors:** Ahmed Alsalem, Bayan Almasoudi, Ghaida Alzahrani, Lama Sindi, Joud Alwan

**Affiliations:** ^1^Department of Ophthalmology, Prince Sultan Military Medical City, Riyadh, Saudi Arabia; ^2^College of Medicine, Umm Al-Qura University, Makkah, Saudi Arabia

## Abstract

We are reporting the case of a 3-year-old-girl who initially presented with unilateral eyelid swelling and ptosis. A diagnosis of acute lymphoblastic leukaemia (ALL) was eventually made based on an orbital incisional biopsy and a bone marrow examination. Historically, orbital involvement had been linked to myeloid leukaemia; however, in lymphoid leukaemia, they are increasingly being implicated and had been reported as the sole presentation of the disease. These findings stress the importance of conducting ophthalmologic assessments in cases diagnosed with ALL in order to prevent delays in proper assessment and treatment. Management options in orbital disease are fortunately not significantly different than well-established treatment protocols.

## 1. Introduction

Leukaemia is described as a malignant proliferation of immature leucocytes which end up replacing the bone marrow, peripheral blood, and organ tissues with blast cells [1]. Four different entities have been recognized and are classified based on the cell precursor type and disease's chronicity. These subtypes include acute lymphoblastic leukaemia (ALL), acute myeloblastic leukaemia (AML), chronic lymphocytic leukaemia (CLL), and chronic myelocytic leukaemia (CML). Cytogenetics and immune phenotyping also play an important role in differentiating between these subtypes [2].

ALL is the most common childhood variant of leukaemia; it generally presents with pancytopenia and organ infiltrates [3]. Despite the high survival rate following diagnosis, it is still a considerable cause of hospitalization and fatality in childhood populations [4].

Involvement of the eye in haematological malignancies can either present as the first manifestation of the disease or be part of a systemic infiltration by neoplastic cells [5]. Extramedullary infiltration involving the orbits caused by myelocytic and myeloblastic leukaemia is well established in the literature [6]. Lymphocytic and lymphoblastic leukaemia, however, have been reported to a lesser extent; this disease pattern is still sporadic and unique [3, 7].

Diagnosis of ocular involvement in ALL carries a poor prognosis and is often delayed, leading to treatment lags and an increase in relapse rates [8]. This raises the need to increase awareness and recognition of this rare disease presentation.

Our case report details the findings of unilateral orbital swelling as the presenting manifestation of ALL in a 3-year-old girl. In 1995, a report discussing eye involvement patterns of leukaemic tumours in patients from Jeddah, Saudi Arabia, was published [9]. To our knowledge, there have been no further studies describing this uncommon disease presentation in Saudi populations. It is our hope that this report adds to the growing literature surrounding this disease and may help in influencing better screening and diagnosing strategies.

## 2. Case Presentation

A 3-year-old Saudi female was brought by her parents to the emergency department (ER) of Prince Sultan Military Medical City in Riyadh, Saudi Arabia with a 1-month history of gradually progressive and painless swelling of the right upper lid which was associated with ptosis. Additionally, the patient had been diagnosed as a case of conjunctivitis and was treated in a primary healthcare clinic with cold compressors, fluorometholone eye drops, and lubricating eye drops as there was no affection of vision on initial presentation. The parents also recalled an upper respiratory tract infection that occurred 1 month prior to her ER visit. Further history was negative for any systemic manifestations, constitutional symptoms, and history of trauma or past surgery. She had an unremarkable family history.

**Figure 1:**
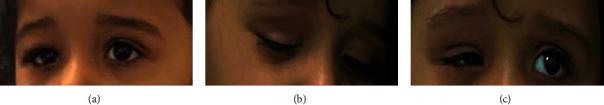
(a–c) Orbital examination for the reported patient. (a) Mild right upper lid swelling. (b) Firm fixed mass at the superior aspect of the orbital rim under the eyebrow; the overlying skin is not erythematous or oedematous but slightly darker in colour. (c) Inferior globe displacement.

On examination, the patient was afebrile and appeared comfortable, alert, and interactive. External examination revealed mild oedema of the right upper lid. The patient was able to fixate her gaze and follow the physician's gestures with both eyes. Pupils were equally reactive without an afferent pupillary defect. Extraocular motility of the right eye was restricted in the superior domain with right inferior globe displacement (Figures 1(a)–1(c)). Motility of the left eye was full. Portable slit-lamp examination was performed. The conjunctiva, cornea, anterior chamber, and lens were normal in both eyes. Dilated fundus examination was normal in both eyes, without evidence of vitreous cells, chorioretinal folds, masses, haemorrhages, or disc oedema.

Apart from the ocular manifestations mentioned earlier, the patient's examination was unremarkable. No lymphadenopathy or hepatosplenomegaly had been found. The patient's mucocutaneous, cardiopulmonary, abdominal, genitourinary, neurological, and musculoskeletal examinations were unrevealing.

Initial laboratory investigations revealed leucocytosis of 20.8 × 10^9^/l with a marked increase in the absolute lymphocytic count to the level of 16.5 × 10^9^/l. Microcytic hypochromic parameters were also found with low normal haemoglobin levels (11.9 g/dl) and a normal platelet count (255 × 10^9^/l). High lactic dehydrogenase (LDH) and ferritin had also been recorded. The C-reactive protein (CRP) was normal (1 mg/l); however, the patient's erythrocyte sedimentation rate (ESR) was elevated (19 mm/hour) according to age-based calculations. Her liver enzymes were unremarkable while the coagulation profile showed a mild prolongation of partial thromboplastin time (PTT). Elevated creatinine level with hypernatremia, hyperkalaemia, hypercalcemia, hypermagnesemia, and hyperphosphatemia were noticed on presentation. Further analysis with a haemoglobin electrophoresis and a quantitative assay suggested an affliction with both sickle cell trait and glucose-6-phosphate dehydrogenase (G6PD).

**Figure 2:**
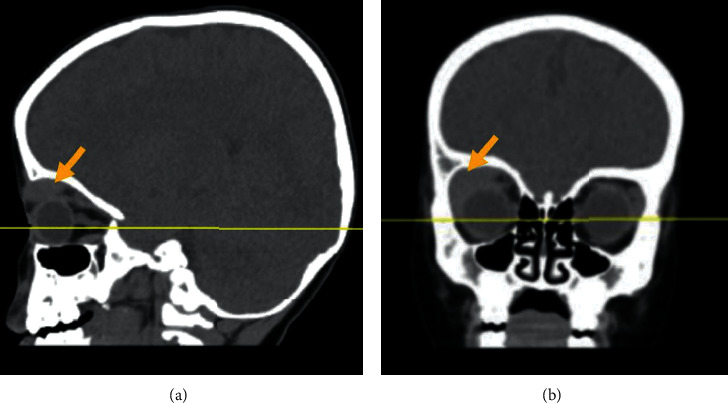
(a, b) A noncontrast computed tomographic scan of the orbits for the reported patient. A noncontrast computed tomographic scan of the orbits demonstrated a well-defined right intraorbital, extraconal, slightly hyperdense lesion noted at the superior and lateral aspect of orbit, abutting the lacrimal gland. It is indenting the superior rectus muscle and eye globe. No defined bone erosion could be seen.

**Figure 3:**
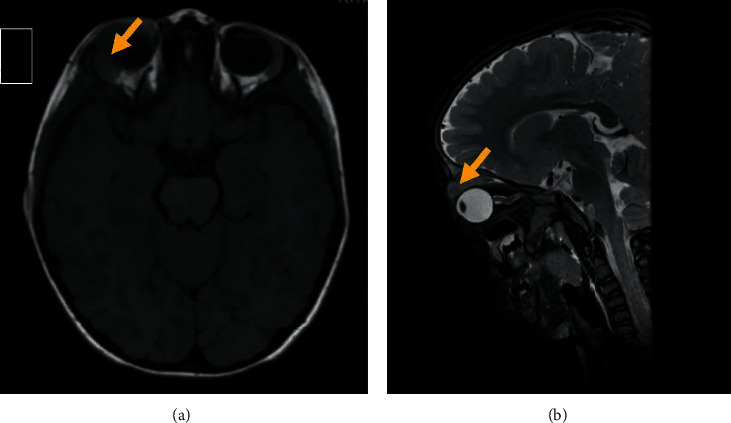
(a, b). Magnetic resonance imaging of the brain for the reported patient. Magnetic resonance imaging of the brain revealed soft tissue T1 and T2 isointense lesion identified in right superior orbit separable from the right lacrimal gland. The lesion appears to be well defined and extraconal. No associated boney erosions or intracranial infiltration was noted. It is indenting and displacing the superior rectus muscle and eye globe. Her optic nerve appeared normal. No brain abnormalities were demonstrated.

A noncontrast computed tomographic scan (CT) of the orbits demonstrated a well-defined right intraorbital, extraconal, and slightly hyperdense lesion noted at the superior and lateral aspects of the orbit, abutting the lacrimal gland and indenting the superior rectus muscle. Magnetic resonance imaging (MRI) of the brain confirmed a soft tissue isointense lesion in the right superior orbit while the optic nerve was intact and no intracranial infiltrations were noted, and further imagining details can be found in Figures 2(a) and 2(b) and Figures 3(a) and 3(b). Lumbar puncture was done as well to rule out central nervous system involvement which revealed a clear, colourless cerebrospinal fluid with undetectable white blood cells and red blood cells.

An incisional biopsy of the mass was performed under general anaesthesia with the biopsy revealing an infiltrative malignant lymphoid neoplasm representing B-cell lymphoblastic leukaemia. The tumour cells are positive for CD45, PAx-5, CD79a, and TdT and negative for CD3, CD20, Desmin, and Synaptophysin.

The patient was referred to the paediatric haematology service. A bone marrow aspirate was done, and a markedly hypercellular marrow was exhibited with marked infiltration by leukaemic blasts approaching around 80% of cells. The hematopoietic cells were markedly reduced. Flow cytometry was done and showed a 70% population of blast cells expressing CD45 (dim), CD10, CD19, CD38, CD34, CD22, TdT, and CD79a with negative expression for CD20, MPO, CD3, and other myeloid T-cell markers.

The patient was subsequently started on chemotherapy including vincristine (1.5 mg/m^2^) at a dose of 0.8 mg intravenous (IV) bolus which was increased to 1 mg IV bolus over 10 minutes in the subsequent 2 days. This was followed by methotrexate intrathecally at a dose of 10 mg, cyclophosphamide (1 g/m^2^) at a dose of 650 mg IV over 1 hour, pegylated asparaginase (2500 IU/m^2^) at a dose of 1625 IU IV over 90 minutes, and cytarabine at a dose of 50 mg IV bolus daily for 1 week. Following this protocol, the patient remained in clinical remission and is following with a paediatric haematologist.

## 3. Discussion

Orbital leukaemic tumours are characterized by their childhood age predominance [7]. These presentations were more commonly associated with AML. An ancient Turkish report published in The Lancet Journal in 1970 hypothesized that childhood myeloid leukaemia tends to be tumorous rather than diffuse and can cause chloroma like ocular mass [10]; furthermore, another Turkish study published in Nature Journal in 2002 suggested that ocular involvement in myeloid leukaemia carries a poor prognosis even in the presence of favourable cytogenetics [11].

Multiple international literature studies suggested a stronger association between orbital infiltration and myeloid leukaemia; in one North American article, out of the 27 patients afflicted with ophthalmic involvement, only 18.5% had ALL compared to 77.8% who had AML and 3.7% who had CML [7]. Another paper from Italy had evaluated 188 leukaemia patients and found that ocular lesions were more frequently seen in patients with AML (66.6%) compared to patients with ALL (15.1%) [12]. Similarly, a larger review of 288 patients from India reported that ocular changes occurred in only 29.2% of lymphoid leukaemia patients compared to more than 41% in myeloid leukaemia patients [13]. In contrast to the abovementioned study, in 1995 a local report from Saudi Arabia studied 17 patients with leukaemia and orbital affection and found that 10 out of the 17 patients (59%) had ALL while 4 patients (23.5%) had AML and 3 patients (17.5%) had CML [9].

Orbital affection may present as the sentinel disease manifestation in up to 10% of leukaemia. In ALL, it is characterized by the possible involvement of all eye components as well as adnexal structures. This occurs either by direct infiltration by leukaemic cells, by other secondary systemic manifestations of the disease, or by therapeutic adverse events [7, 14].

Bidar et al. reported that leukaemia was eventually diagnosed in around 11% of children who initially presented with unilateral ptosis [7]. Given the wide range of structures involved, these lesions may manifest in a myriad of different ways, from asymptomatic presentations to proptosis and orbital swelling which may be unilateral, bilateral, painful or even painless to chemosis, diplopia, decreased vision, or blindness [14–17]. A formal ophthalmologic examination in all patients with leukaemia is prudent.

In contrast to our patient who presented with an elevated leukocytic count, low normal haemoglobin, and a normal platelet count, multiple reports discussing the haematological findings in leukaemic ophthalmopathies showed an association between elevated leukocytic count in AML. ALL ocular involvement that was associated with anaemia and thrombocytopenia also put the patients at a greater risk of developing intracranial haemorrhages [1, 18, 19].

Leukaemic orbital masses appear as homogenous lesions that are isointense to brain and muscle or slightly hyperdense in CT imaging with mild enhancement if contrast media was used. In MRI images, they appear isointense to grey matter and muscle and are usually either poorly enhancing or nonenhancing. As in our patient, invasion of adjacent structures often occurs in a lateral predilection. However, bony destruction is not a commonly found behaviour [7].

Other than leukaemias, orbital lesions can be associated with rhabdomyosarcoma, which is considered among the commonest primary orbital neoplasm in children; other differential diagnoses include lymphoma, specifically Burkitt's lymphoma, nerve sheath tumour like optic nerve glioma, and other neoplasms from osseous origin like dermoid cyst, osteosarcoma, and neuroblastoma [20].

Both primary and relapsed leukaemia might present with ocular involvement preceding systemic diseases; thus, combined histopathological evidence from the orbital tissue and bone marrow is needed in order not to delay the treatment, which is usually not significantly distinctive in comparison with protocols given to leukaemia patients without ophthalmopathies. Furthermore, a lumbar puncture is often necessary to exclude central nervous system (CNS) involvement [6, 21].

Due to established prophylactic CNS protocols in patients with ALL, the incidence of orbital involvement has decreased in the past few decades. The occurrence of this manifestation however carries a worse prognosis and an increased morbidity and mortality rate. Thus, early systemic chemotherapy with possible radiotherapy is needed to preserve the vision and improve survival. These treatment modalities have their own adverse effects on orbital structures [22, 23], which necessitate a multidisciplinary team approach between an ophthalmologist, a haemato-oncologist, and a radiation-oncologist.

In conclusion, we report the case of a 3-year-old-girl who was diagnosed with ALL based on an orbital biopsy after an insidious presentation of unilateral eye swelling and ptosis. Despite this manifestation not being significantly different in terms of cytogenetics and immunophenotyping analysis or treatment protocols, it carries a poorer prognosis and needs more dedicated attention by the ophthalmologist with prompt referral to specialized services as this particular presentation is increasingly being reported as the initial manifestation of leukaemic tumours.

## Data Availability

Further laboratory and radiological data used to support the findings of this study are restricted by Prince Sultan Military Medical City, Riyadh, Saudi Arabia, in order to protect Patient Privacy. Data are available from Ahmed Alsalem, asalsalem@psmmc.med.sa, for researchers who meet the criteria for access to confidential data.
